# British Sheep Breeds as a Part of World Sheep Gene Pool Landscape: Looking into Genomic Applications

**DOI:** 10.3390/ani11040994

**Published:** 2021-04-01

**Authors:** Michael N. Romanov, Natalia A. Zinovieva, Darren K. Griffin

**Affiliations:** 1 School of Biosciences, University of Kent, Canterbury, Kent CT2 7NJ, UK; D.K.Griffin@kent.ac.uk; 2 L.K. Ernst Federal Research Center for Animal Husbandry, Dubrovitsy Estate, Podolsk District, Moscow Region, 142132 Podolsk, Russia; n_zinovieva@mail.ru

**Keywords:** British sheep breeds, conservation, adaptation, disease resistance, phenotypic traits, genetic improvement, genomic research, SNPs, QTLs, whole genome sequence

## Abstract

**Simple Summary:**

The UK can be proud of the fact that numerous native breeds of sheep have been developed here that possess unique phenotypic features and excellent productivity and are utilized throughout the world. Their remarkable popularity and further sustainable breeding on grass pastures of British Isles and elsewhere can benefit from genomic applications.

**Abstract:**

Sheep farming has been an important sector of the UK’s economy and rural life for many centuries. It is the favored source of wool, meat and milk products. In the era of exponential progress in genomic technologies, we can now address the questions of what is special about UK sheep breed genotypes and how they differ genetically form one another and from other countries. We can reflect how their natural history has been determined at the level of their genetic code and what traces have been left in their genomes because of selection for phenotypic traits. These include adaptability to certain environmental conditions and management, as well as resistance to disease. Application of these advancements in genetics and genomics to study sheep breeds of British domestic selection has begun and will continue in order to facilitate conservation solutions and production improvement.

## 1. Introduction

Sheep are an integral element of UK countryside landscapes at a range of altitudes, with both modern and ancient breeds (i.e., specific domestic animal groups of a particular origin and with homogeneous phenotype) represented (Table 1 and Table S1). Their ubiquity in the scientific literature dates back at least to William Youatt [1] (Figure S1) who provided an impressive overview of sheep breeding, management, veterinary aspects and their relevance to everyday life.

The UK is a largest producer of sheep than any EU country, counting nearly 34 million adult animals and lambs [13]. Prior to Brexit, this constituted a quarter of the EU sheep population and 3% of the global flock. The UK sheep-meat sector occupies the sixth place in the world and produces more than one third of European sheep meat. Overall, sheep farming provides the employment economy with the equivalent of £291M per annum [5]. This important livestock production sector is targeted by the biennial Sheep Breeders Round Table [14], a major event held by the National Sheep Association every two years.

A glance at the gene pool landscape for British sheep breeds from the National Sheep Association leads to a list of over 60 native breeds [7], whereas there are over 1400 discrete breeds throughout the world [15,16]. Other reports state that the UK has around 90 native sheep breeds and crossbreeds, more than any other nation [5]. They are bred for meat, milk, wool as well as vegetation management/recreation purposes. Traces of breed-specific artificial selection for phenotypic (qualitative and quantitative) traits are retained in the genome of each breed. Relevant examples of genetic and genomic applications and implications will be overviewed here, with a special focus on British sheep.

## 2. Geographical Distribution

### 2.1. British Isles

**Figure 1:**
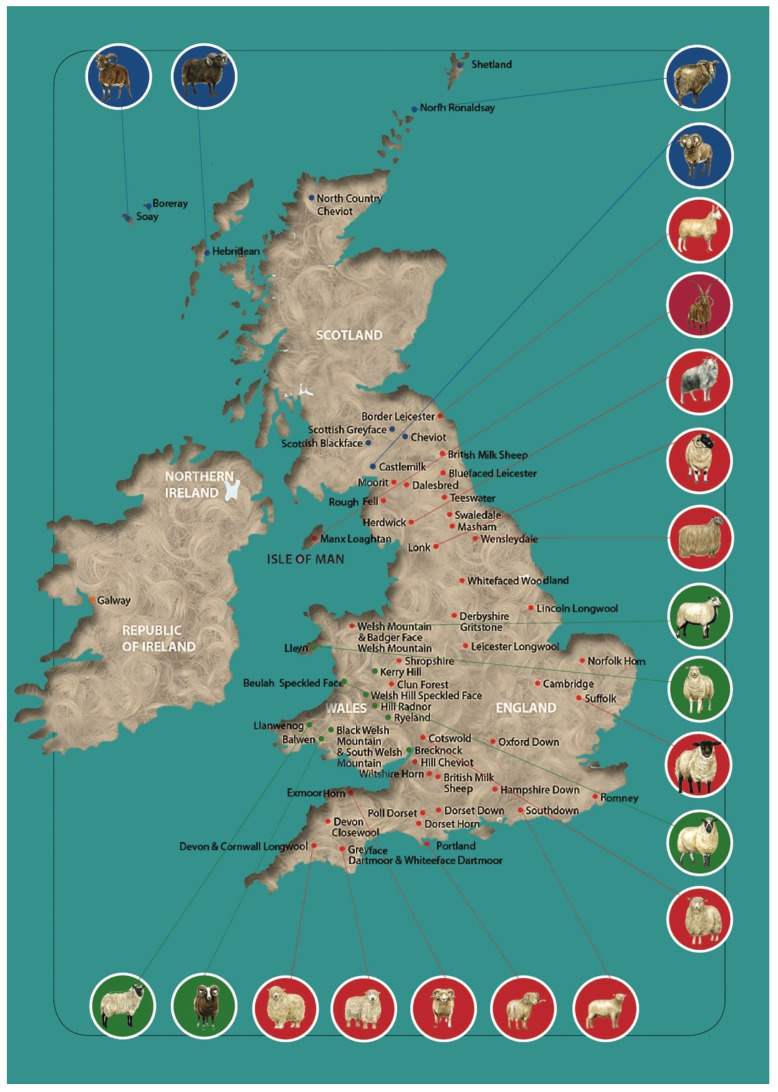
Geographical distribution of native sheep breeds in the British Isles. Modified from [5,6].

Sheep breed gene pool of British Isles can be subdivided into geographical groups (Figure 1) according to their origin from England (Border Leicester; Clun Forest; Dorset Horn, Figure S1d,e; English Leicester, Figure S1h,i; Romney; Southdown, Figure S1l,m; Suffolk; Wiltshire Horn; etc.), Isle of Man (Manx Loaghtan), Scotland (Cheviot, Figure S1b,c; Scottish Blackface, Figure S1a; etc.), Wales (Badger Face Welsh Mountain, Balwen Welsh Mountain, Beulah Speckled Face, Black Welsh Mountain, Brecknock Hill Cheviot, Hill Radnor, Improved Welsh Mountain, Improved Welsh Mountain, Kerry Hill, Llandovery Whiteface, Llanwenog, Lleyn, Talybont Welsh, Welsh Hardy Speckled Faced, Welsh Mountain-Hill Flock, etc.), and Ireland (Galway). Major breeds of British Isles are listed in Table 1, with more details on origin and description of selected sheep breeds being provided in Table S1.

The geographical concentration (endemism) of the British sheep breeds was suggested to be a major risk factor for breed endangerment [17].

### 2.2. Outside Britain

British sheep breeds are common in other parts of the world and have been exported from the UK to other countries to create new breeds and improve the extant ones, with Australian, USA and European breeds being prominent examples [4,18]. As in other examples, imports into the former USSR can be mentioned when British breeds served as a basis for developing new breeds, e.g., Gorky and Russian Long-wool [19,20,21,22,23,24]. In particular, British meat sheep breeds have had a significant impact on the development of native gene pool breeds for semi-fine sheep production in Russia and former USSR countries in the last century [25,26,27,28,29] (see Supplementary Information S1, Table S2 and Figure S2).

## 3. Phenotypic Diversity

Many of British breeds are unique in terms of phenotypic traits and adaptation to local conditions [30] (see examples in Table S1) but nonetheless may contribute to the commercial herds. For instance, Bluefaced Leicester is believed to be most distinguished breed in the UK as it sires almost half of the commercial hybrid progeny [5].

In terms of peculiar adaptability, specific phenotypes and features of the British sheep breeds, some of them are lowland breeds (Suffolk, Wiltshire Horn), some are hill breeds (Cheviot, Scottish Blackface, Brecknock Hill Cheviot, Hill Radnor, Welsh Mountain-Hill Flock), and some are upland breeds (Beulah Speckled Face, Llandovery Whiteface, Llanwenog), while some others are adapted to variable conditions (Clun Forest, Badger Face Welsh Mountain, Kerry Hill, Lleyn) [7]. An extraordinary example of adaptation is the North Ronaldsay breed localized on an Orkney island, whose copper-deficient and high-sodium diet is based predominantly on the seaweed *Laminaria* [31].

Body size varies from small (Black Welsh Mountain) to medium (Clun Forest, Llanwenog, Lleyn, Welsh Mountain-Hill Flock) to large (Border Leicester, Romney, Wiltshire Horn, Talybont Welsh). Some breeds are horned (Dorset Horn, Wiltshire Horn, Scottish Blackface), others are hornless (Border Leicester, Romney, Beulah Speckled Face, Kerry Hill, Lleyn), and in some others only males have horns (Cheviot, Black Welsh Mountain, Balwen Welsh Mountain, Brecknock Hill Cheviot, Llandovery Whiteface, Welsh Mountain-Hill Flock). The Border Leicester breed has a characteristic “Roman nose”.

Most breeds are used for producing lamb meat, while some (Clun Forest) are dual purpose and can be used for meat, wool and milk. There are long-woolled breeds (e.g., Romney whose fleece is suitable for carpets), and others (e.g., Wiltshire Horn) naturally shed wool, not requiring shearing. Females of some breeds (Dorset Horn, Hill Radnor) have a slightly shorter gestation period allowing 1.5 lambs a year. Some breeds (Romney, Balwen Welsh Mountain, Lleyn) have a low disease risk. Previously, British sheep breeds and flocks were assessed, on the base of prion protein (PrP) genotypes, for risk of scrapie, also known as prion disease and transmissible spongiform encephalopathy [32,33,34,35], and its atypical form [36] that had implications for breeding programs.

## 4. Conservation Issues: To Breed or Not to Breed

Over several decades, there has been a general trend in the world’s animal production to substitute or cross local breeds with productive commercial stocks that may lead to genetic diversity reduction [18]. Such an animal breeding practice puts native breeds at risk of extinction, even prior to characterization of their variation, signatures of adaptations and breed history at the genomic level (e.g., [37,38,39,40]).

The Rare Breeds Survival Trust names 57 British native sheep breeds, including 25 breeds on the critical list, e.g., Border Leicester, Dorset Horn, Balwen Welsh Mountain, Hill Radnor and Llanwenog [9]. Indeed, most British sheep breeds, especially the rare ones, have long history and noteworthy ancestry [1,2,3], with some of them being dated 1st century (Badger Face Welsh Mountain) or brought by Romans (Wiltshire Horn). Some others have been known since the Middle Ages (Romney, Scottish Blackface, Cheviot, Black Welsh Mountain, Welsh Mountain-Hill Flock). With this in mind, there is a desperate need to develop appropriate sheep breed conservation strategies.

The collection of breed data including adaptation to specific habitats facilitates development of conservation solutions. Characterization of genetic originality and authenticity of a breed via its DNA analysis is a crucial component in informed decision making to decide whether a breed (or population) is worthy or not of conservation action. Information regarding the risk status and the level of inbreeding can be further used to choose respective conservation strategies (in situ vs. ex situ, pure breeding vs. crossbreeding, etc.). Novel molecular and advanced genomic tools can be effectively employed to incorporate genetic variability and genomic information into conservation planning and sustainable conservation programs [4,41]. Characterized, conserved and maintained genetic diversity of native breeds can be further used for adaptation to local environments, future change in breeding requirements, and as a source of research material (e.g., [42,43]).

## 5. Sheep Breed Diversity and Genomic Research

At present, there is a rich arsenal of genetic and genomic resources, tools and applications used for livestock assessment, breeding and production including, first of all, genetic profiling of diverse breeds, and search for quantitative trait loci (QTLs) and candidate genes in farm animals. These genomic advances facilitate breed improvement and understanding of the genetic processes in the course of domestication and breed evolution. For example, the Dorset breed was found to be a carrier of a single polymorphism mutation at the callipyge (*CLPG*) locus causing the muscle hypertrophy phenotype [44].

It requires a major undertaking to evaluate genetically most widespread industrial breeds [39], such as the Texel in sheep [45]. However, more and more attention is being drawn to surveying and analyzing local livestock breeds. This is due to their adaptive properties, as reflected in their genomic structure, and their potential to improve performance, resistance and environment impact of commercial herds (e.g., [10,37,39]).

### 5.1. Genetic Diversity, QTL and Candidate Gene Characterization

To characterize genetic structure and diversity in the sheep, various molecular markers were previously utilized, including microsatellites (e.g., [46,47,48,49,50]; see for review [30,51]), mtDNA (e.g., [51,52,53]) and endogenous retroviruses [30,50,54]. For example, in a study of three English breeds [30,50], it was shown that they were clearly distinguished relative to one another for ten microsatellite loci. One breed, the Herdwick, was unique for high frequency of the R0 retrotype indicative of a primitive genome that is absent in the mainland UK breeds and known only for few other non-British breeds.

Using microsatellite markers, QTLs associated with muscle depth were characterized in British commercial terminal sire sheep including the Suffolk breed [55]. One QTL for muscle depth was verified in Suffolk sheep on chromosome 1.

Since ewe prolificacy was associated with certain mutations in the *BMP15* and *GDF9* candidate genes, it was explored in UK and Ireland sheep by their genotyping for these alleles [56]. Three mutations had large effects on ovulation rate in the Cambridge and Belclare (of Irish origin) breeds, with two alleles being transferred from the Lleyn breed (of Welsh origin) and one from a High Fertility line in Ireland.

Genetic resistance to nematode infection is an important target of selective breeding for this trait in the UK. This was studied within a purebred Scottish Blackface flock by partial resequencing genes in the Major Histocompatibility Complex (MHC) class II region [57]. Causal mutant alleles at the *DRB1* and *DQB2* loci were identified that were associated with this trait. Single nucleotide polymorphisms (SNPs) in three other candidate genes for nematode resistance and body weight were examined in populations of domestic Scottish Blackface and free-living Soay ewe lambs, and a nominally significant association between an *IL23R* SNP and body weight was found [58].

Other examples of candidate genes, for example, associated with ewe mature weight are *TMEM8B* and *SPAG8* that showed picks of a signature of selection at single SNPs in four sheep breeds, the Suffolk among them [59].

### 5.2. Genomic Applications

With the advent of next generation sequencing (NGS) technologies, SNP panels and a whole genome sequence draft became available for the sheep by 2010 [15,60] that can also be used for querying genomic features of British breeds. The remarkable milestone in this field was the annotated sheep genome sequence Oar v3.1 published in 2014 [61]. Another improved assembly, Oar_v4.0, was produced using PBJelly 2 software [62]. The latest genome assemblies were generated in 2017 and 2020, and designated Oar_rambouillet_v1.0 (sheep reference genome; [63]) and ASM1117029v1 [64], respectively.

These state-of-the-art resources are crucial for genetic improvement of the existing sheep flock by implementing genome-wide association studies (GWAS; e.g., [45]), analysis of quantitative traits and genomic selection [65]. However, a key prerequisite for these applications is a thorough examination of genetic structure and variation within and between sheep breeds including the British ones. This information also helps elucidate domestication pathways, breed formation and population history [15]. In particular, insight into demographic history of breeds can provide a set of genetic markers for obtaining individual genomic estimated breeding values (GEBV) (i.e., genomic selection) and their applicability to other populations [10]. Efficacy of genomic and marker-assisted selection, and QTL spotting via GWAS depends on knowledge of population structure and origin [10].

After marker validation, genetic or genomic selection is feasible when targeting, for example, such sheep traits as footrot resistance [45] and mature body weight [59]. For genomic selection implementation, a genotyped reference population is built for GEBV evaluation. As low heritability and polygenic nature is inherent in selected quantitative traits, genomic selection hopefully improves selection response if compared to conventional best linear unbiased prediction-assisted selection [45].

There are two major collaborative sheep genomics groups, the International Sheep Genomics Consortium [60] and an Australia- and New Zeeland-based project, SheepGenomesDB [66]. Another beneficiary group is the Ovine Functional Annotations of Animal Genomes (FAANG) Project [11,67,68]. Studies within the framework of the FAANG [68,69] and related projects [70] also produced sheep genome datasets including those for British breeds.

#### 5.2.1. SNPs

Use of multiple SNP markers has substantially enhanced analysis of genetic diversity and population history [30,52,71,72,73,74], especially thanks to the sheep HapMap project [10,12,15,60,75,76,77]. For instance, in a genome-wide survey of SNP variation [15], it was demonstrated that the British Suffolk genetically differentiated from two American Suffolk subpopulations, whereas the genetic structure of Australian Poll Dorset and American Dorsets was also different. In another research of genetic structure and admixture in terminal sire breeds in the USA using Applied Biosystems Axiom Ovine Genotyping Array (50K) and Illumina Ovine SNP50 BeadChip, the Suffolk, Hampshire, Shropshire and Oxford (terminal) sheep were genotyped along with the Rambouillet (or the French Merino; dual purpose) sheep [78]. There was a clear-cut divergence between the Suffolk sheep from two different US regions. The Hampshire, Suffolk, and Shropshire breeds demonstrated the greatest admixture. Relative to sheep from other world regions, the US terminal breeds of British origin formed a separate cluster suggesting their genetic distinctiveness.

The earliest research of SNP-based diversity in UK sheep showed genetic distinctiveness of three English native hill breeds examined at three SNP loci associated with phenotypes [30,50]. In a broader study of 18 Welsh local breeds as a selected segment of the UK’s sheep germplasm [10], the Illumina OvineSNP50 array was employed to examine genetic structure of these breeds. A similar methodology was exploited to elucidate genetic diversity and genome selection in the Suffolk, Rambouillet and three Rambouillet-related breeds from the USA [79]. The Suffolk sheep were clearly distinguished from the four others in terms of diversity and differentially selected genome regions.

SNPs have also become genetic markers of choice in searching for QTLs and conducting GWAS in sheep (e.g., [45,80,81,82,83,84]). The Illumina OvineSNP50 chip was utilized for a GWAS and regional heritability mapping (RHM) to identify QTLs for nematode resistance and body weight in Scottish Blackface lambs [85]. Strong associations were found on chromosomes 14 and 6 for nematode resistance, and on chromosome 6 for body weight. An additional RHM study in three European populations (including Scottish Blackface) revealed other QTLs for nematode resistance, with one on chromosome 20 being the most significant and located close to MHC, as a functional candidate for this trait [86]. In the follow-up investigation [87], accuracy of genomic prediction within and across populations for nematode resistance and body weight was assessed in two British purebred (Scottish Blackface, British Texel) and two non-British backcross populations. Genomic estimated breeding values (GEBV) were definitely better within populations that points out a more accurate genomic prediction in closely related sheep than across breeds. Later, using a 932-SNP assay, an independent validation search for nematode resistance QTLs in three sheep breeds (including Scottish Blackface and Suffolk) suggested that inconsistency of SNP effects may occur in different populations [88].

The same Illumina OvineSNP50 genotype panel was an effective tool for investigating runs of homozygosity (ROHs) and selection signatures in six commercial European meat breeds including the Suffolk sheep (of Irish population) [89]. The Suffolk breed showed a distinct population structure different from five other breeds. Moreover, the Suffolk sheep were the least admixed, although they formed two non-overlapping clusters, one of them being a subpopulation of New Zealand origin. The Irish Suffolk population was more abundant in ROHs, suggesting its smaller effective population size both in recent and past generations, and a higher relatedness among this breed. The Suffolk (along with the Beltex) had the largest number of putative selective sweeps.

#### 5.2.2. Whole Genome Sequencing

Further development of NGS platforms and reduction of their cost make it possible to implement whole genome sequencing for numbers of individuals within one or more species. Whole genome sequences seem to provide ultimate evaluation of genetic variability and candidate mutations that can be further used for genome-wide association studies, sequence genotype imputation and genomic prediction improvement as a component of genomic selection [65,66].

Using whole genome sequences of 21 Chinese native sheep breeds, Yang et al. [90] identified candidate genes, pathways and gene ontology categories presumably related to high-altitude and arid environments.

Naval-Sanchez et al. [70] sequenced 43 worldwide sheep breeds and functionally annotated their whole genome sequences, demonstrating that selection sweeps correspond to coding genes, proximal regulatory elements and active transcription sites, and suggesting that remodeled gene expression could play an important evolutionary role in sheep breed diversification.

On the basis of whole genome sequences for 145 wild and domestic sheep and goat samples, Alberto et al. [72] found selective sweeps that led to domestic breed divergence as well as genomic signatures for convergent domestication in two related species.

Using SNP and whole genome sequence data for a large worldwide sample collection of wild and domestic animals, Chen et al. [77] found that in sheep there might be an accelerated genetic drift vs. reduced directional selection on X chromosome as compared to autosomes.

The ongoing Australia- and New Zeeland-based project SheepGenomesDB is aimed at sequencing 453 (Run1) and 935 (Run2) animals, which is supposed to cover a global sheep breed diversity in order to identify causative mutations and facilitate genomic selection [65,66].

### 5.3. British Sheep Genome Studies

To date, there has not been launched an overall, comprehensive genetic/genomic survey of the British sheep gene pool. Certain British native breeds have been part of either diversity studies using different molecular markers or whole genome sequences and often a limited number of samples per breed. For example, using the Illumina HiSeq 2000 platform, three individual animals, each representing one Welsh sheep breed (Hardy Speckled Faced, Dollgellau and Tregaron Welsh Mountain), were sequenced in order to explore their demographic history [10].

Recently, more whole genome sequences were generated for separate British breeds. For instance, the Illumina whole genome sequences for seven Cambridge sheep and one British du Cher individual were obtained in a comparative study including also the Romanov and two Iranian breeds [91]. The Cambridge breed was genetically different, while the British du Cher being close to the Romanov breed. A higher number of short ROHs was detected in the Cambridge sheep and a lower number of long ROHs in the British du Cher, meaning a lower recent inbreeding in the latter breed.

Whole genome sequences of 17 Poll Dorset sheep was compared to those from two Tibetan breeds [92]. Selection signatures were identified that include candidate genes putatively associated with hypoxia responses, meat traits and disease resistance.

There are also international projects that have generated whole genome sequences for British and non-British breeds and can be served as data sources for further studies [69,89,93,94]. In a recent global genomic survey of wild sheep and domestic breeds [95], ten Suffolk and seven Shetland individual whole genomes were included and resequenced for seeking important gene associations with morphological and economic traits. One of such iconic traits is tail configuration, and the Shetland breed was used as an example of short thin-tailed sheep. A number of selective sweeps were identified that overlapped with functional genes involved in fat deposition and hair growth. Differences in allele frequencies between fat- and thin-tailed breeds (including Shetland) were found at genes *PDGFD*, *XYLB*, *TSHR*, and *SGCZ*, with *PDGFD* (platelet derived growth factor D) being a specially remarkable candidate for fat deposition in tail.

## 6. Conclusions

The UK sheep landscape is characterized by abundant breed history and genetic diversity, suggesting its preservation for sustainable use and advanced research. The latter can rely upon state-of-the-art molecular and genomic tools including SNPs and whole genome sequencing. This opens further opportunities to elucidate breed ancestry, variability, and genetic merit of particular markers and candidate genes for adaptation, genomic selection and production improvement. Further research is anticipated to understand comprehensively how genomic landscape of the British sheep has contributed to the fact that over the centuries they have thrived to current numbers and value, delighting our eyes on the green landscapes of today’s Great Britain.

## Figures and Tables

**Table 1:** List of major sheep breeds of British Isles.

Name/Subtype	Alternative Name	Region/Country	Purpose	References
Northern European short-tailed sheep (group of breeds)	—	British Isles, Scandinavia, Germany, Russia	meat, milk, wool	[2,3]
Bluefaced Leicester	—	England	meat	[4,5,6,7]
Border Cheviot/Cheviot subtype	South Country Cheviot	England	meat	[8]
Border Leicester	—	England	meat	[2,4,5,6,7,9]
British Milksheep	—	England	milk	[7]
Cambridge	—	England	meat, milk	[1,7]
Clun Forest	—	England	meat, milk, wool	[1,4,7,10]
Cotswold	—	England	meat, wool	[1,2,4,5,6,7,9]
Dalesbred	—	England	meat, wool	[2,7]
Derbyshire Gritstone	—	England	meat	[1,2,7,9]
Devon Closewool	—	England	meat	[2,7,9]
Devon Longwoolled	Devon Longwool, Devon and Cornwall Longwool	England	meat, wool	[1,2,5,6,7,9]
Dorset	Dorset Horn, Dorset Horned	England	meat	[1,4,9]; Figure S1d,e
Dorset Down	—	England	meat	[1,2,7,9]
Easy Care	—	England	meat	[7]
English Leicester	Bakewell Leicester, Dishley Leicester, Improved Leicester, Leicester, Leicester Longwool, New Leicester	England	meat	[1,2,4,6,9]; Figure S1h,i
Exlana	—	England	meat	[7]
Exmoor Horn	—	England	meat	[1,5,7]
Greyface Dartmoor	—	England	meat	[1,5,9]
Hampshire	Hampshire Down	England	meat	[1,4,7]
Herdwick	—	England	meat	[1,5,6,7]
Lincoln	Lincoln Longwool	England	wool	[1,4,9]
Lonk	Improved Haslingden	England	meat, wool	[5,7,9]
Masham	—	England	meat, wool	[5,7]
Meatlinc	—	England	meat	[7]
Norfolk Horn	Blackface Norfolk Horned, Norfolk Horned, Old Norfolk, Old Norfolk Horned	England	meat	[1,2,4,6,7,9]; Figure S1k
North of England Mule	—	England	meat, milk	[7]
Oxford	Oxford Down	England	meat	[2,4,5,7,9]
Portland	—	England	meat	[1,4,5,6,9]
Romney	Romney Marsh, Kent	England	meat, wool	[1,2,4,6,7]; Figure S1g
Rough Fell	—	England	meat	[2,4,7]
Ryeland	—	England	meat	[1,2,6,7]; Figure S1n
Shropshire	—	England	meat	[1,2,6,7]
Southdown	—	England	meat	[1,2,4,5,6,7]; Figure S1l,m
Suffolk	—	England	meat	[1,2,4,5,6,7]
Swaledale	—	England	meat, wool	[2,7]
Teeswater	—	England	meat	[1,2,4,6,7,9]; Figure S1o
Wensleydale	—	England	meat	[5,6,7,9]
Whiteface Dartmoor	—	England	meat	[5,7,9]
Whitefaced Woodland	Penistone	England	meat	[4,6,7,9]
Wiltshire Horn	—	England	meat	[1,2,4,7]
Galway	—	Ireland	meat	[1,4,6,10]
Manx Loaghtan	Loaghtyn, Loghtan	Isle of Man	wool	[5,6,7,9]
Boreray	Boreray Blackface, Hebridean Blackface	Scotland	meat	[4,5,6,7,9]
Bowmont	—	Scotland	meat, wool	[4,6]
Castlemilk Moorit	Castlemilk Shetland, Moorit Shetland	Scotland	hobby	[5,6,7,9]
Cheviot	—	Scotland	meat, wool	[1,7]; Figure S1b,c
Hebridean	St Kilda	Scotland	vegetation management	[1,3,5,6,7]
North Country Cheviot/Cheviot subtype	—	Scotland	meat	[7]
North Ronaldsay	Orkney	Scotland	wool	[1,3,5,6,9]
Scotch Mule	—	Scotland	meat	[7]
Scottish Blackface	Blackfaced Highland, Kerry, Linton, Scotch Blackface, Scotch Horn, Scottish Highland, Scottish Mountain	Scotland	meat	[1,2,7,11]; Figure S1a
Scottish Dunface	Scottish Tanface, Old Scottish Short-wool	Scotland (extinct)	meat, wool	[1,3]
Soay	—	Scotland	meat	[2,3,4,5,6,7,9,12]
Shetland	—	Shetland Islands	meat, wool	[1,6,7]
Badger Face Welsh Mountain/Welsh Mountain subtype	Defaid Idloes, Badger Faced Welsh Mountain, Welsh Badger-faced	Wales	meat	[5,7,10]
Balwen Welsh Mountain/Welsh Mountain subtype	—	Wales	meat	[5,6,7,9,10]
Beulah Speckled Face	—	Wales	meat	[5,10]
Black Welsh Mountain/Welsh Mountain subtype	Defaid Mynydd Duon	Wales	meat	[4,6,7,10]
Brecknock Hill Cheviot/Cheviot subtype	Brecon Cheviot, Sennybridge Cheviot	Wales	meat	[1,7,10]
Epynt Hardy Speckled Face	—	Wales	meat	[7]
Hill Radnor	—	Wales	meat	[1,2,7,9,10]
Kerry Hill	—	Wales	meat	[2,7,10]
Llanwenog	—	Wales	meat	[4,5,6,7,9,10]
Lleyn	Dafad Llŷn	Wales	meat	[5,6,7,10]
Nelson South Wales Mountain	—	Wales	meat	[7]
South Wales Mountain/Welsh Mountain subtype	—	Wales	—	[10]
Welsh Halfbred	—	Wales	meat, milk	[7]
Welsh Hill Speckled Face	—	Wales	meat	[7]
Welsh Mountain	Defaid Mynydd Cymreig, Welsh Mountain-Pedigree	Wales	meat	[7,9]
Welsh Mule	—	Wales	meat, milk	[6,7]

## Data Availability

This review did not report any data.

## Supplementary Materials

The following are available online at https://www.mdpi.com/article/10.3390/ani11040994/s1, Figure S1: Old British sheep breeds as shown in the 1837 treatise by William Youatt [1], Figure S2: Examples of Soviet sheep breeds produced using British breeds and presented at the All-Union Agricultural Exhibition (AUAE), Table S1: Origin data and phenotypic characteristics of selected British sheep breeds, Table S2: Native sheep breeds from Russia and former USSR developed using British breeds, Supplementary Information S1: British sheep breeds outside the UK: examples from Russia and former USSR.
