# Supplementary material for: British Sheep Breeds as a Part of World Sheep Gene Pool Landscape: Looking into Genomic Applications
Source: Animals (Basel). 2021 Apr 1;11(4):994. doi: 10.3390/ani11040994 (PMC8103502; doi:10.3390/ani11040994)
Supplement: Supplementary file 1 [file animals-11-00994-s001.pdf]

animals

Supplementary Materials

British Sheep Breeds as a Part of World Sheep Gene Pool Landscape: Looking into Genomic Applications

Michael N. Romanov [1,*], Natalia A. Zinovieva [2] and Darren K. Griffin [1]

1 School of Biosciences, University of Kent, Canterbury, Kent, CT2 7NJ UK; D.K.Griffin@kent.ac.uk
2 L.K. Ernst Federal Research Center for Animal Husbandry, Dubrovitsy Estate, Podolsk District, Moscow Region, 142132 Podolsk, Russia; n_zinovieva@mail.ru
* Correspondence: m.romanov@kent.ac.uk

Simple Summary: The UK can be proud of the fact that numerous native breeds of sheep have been developed here that possess unique phenotypic features and excellent productivity and are utilized throughout the world. Their remarkable popularity and further sustainable breeding on grass pastures of British Isles and elsewhere can benefit from genomic applications.

Citation: Romanov, M.N.; Zinovieva, N.A.; Griffin, D.K. British Sheep Breeds as a Part of World Sheep Gene Pool Landscape: Looking into Genomic Applications. *Animals* **2021**, *11*, 994. https://doi.org/10.3390/ani11040994

Academic Editors Emilia Bagnicka and Aldona Kawęcka

Received: 16 March 2021
Accepted: 29 March 2021
Published: 5 April 2021

Publisher's Note: MDPI stays neutral with regard to jurisdictional claims in published maps and institutional affiliations.

Copyright: © 2021 by the authors. Licensee MDPI, Basel, Switzerland. This article is an open access article distributed under the terms and conditions of the Creative Commons Attribution (CC BY) license (http://creativecommons.org/licenses/by/4.0/).

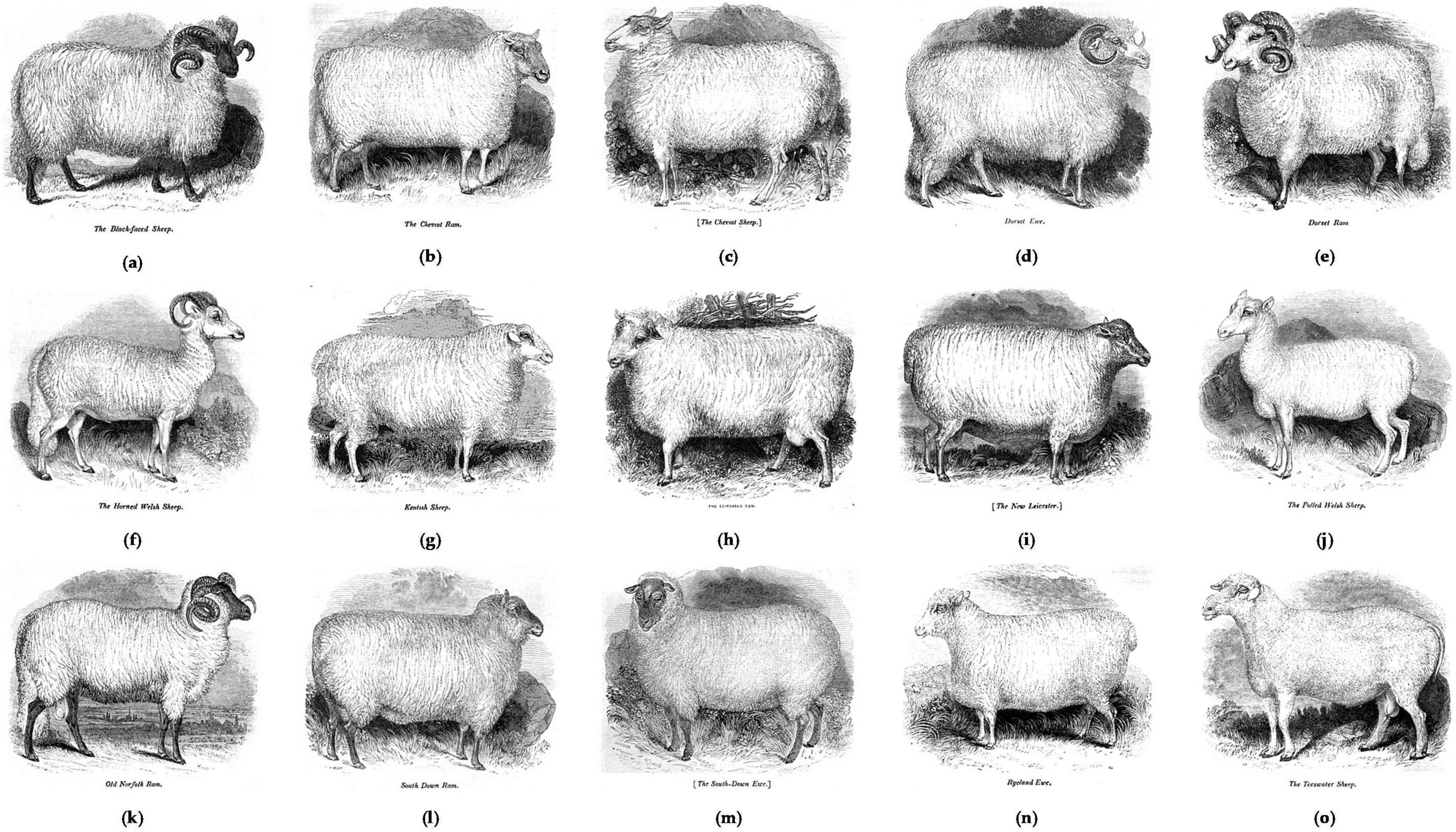
The Black-faced Sheep.
(a)
The Cheviot Ram.
(b)
[The Cheviot Sheep.]
(c)
Dorset Ewe.
(d)
Dorset Ram
(e)
The Horned Welsh Sheep.
(f)
Kentish Sheep.
(g)
(h)
[The New Leicester.]
(i)
The Polled Welsh Sheep.
(j)
Old Norfolk Ram.
(k)
South Down Ram.
(l)
[The South-Down Ewe.]
(m)
Ryeland Ewe.
(n)
The Teeswater Sheep.
(o)

Figure S1. Old British sheep breeds as shown in the 1837 treatise by William Youatt [1]: (**a**) Blackfaced sheep, (**b**) Cheviot ram, (**c**) Cheviot sheep, (**d**) Dorset ewe, (**e**) Dorset ram, (**f**) Horned Welsh sheep, (**g**) Kentish sheep, (**h**) Leicester ram, (**i**) New Leicester, (**j**) Polled Welsh sheep, (**k**) Old Norfolk ram, (**l**) South Down ram, (**m**) South Down ewe, (**n**) Ryland ewe, and (**o**) Teeswater sheep.

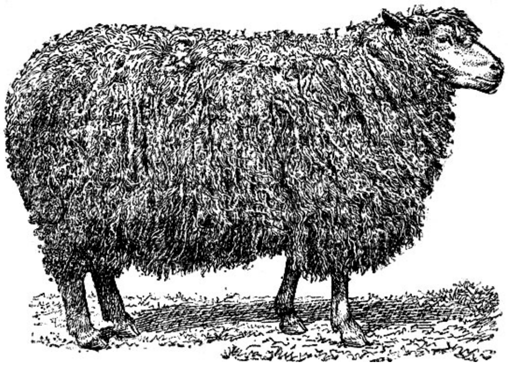

(**a**)

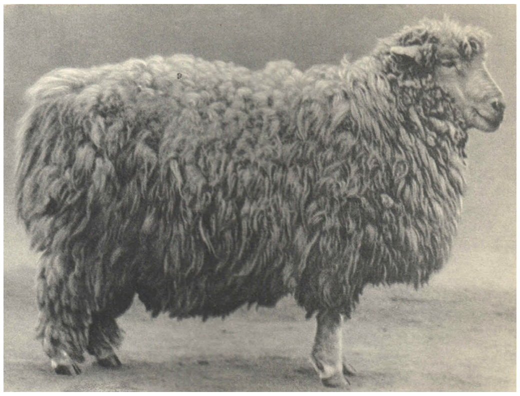

(**b**)

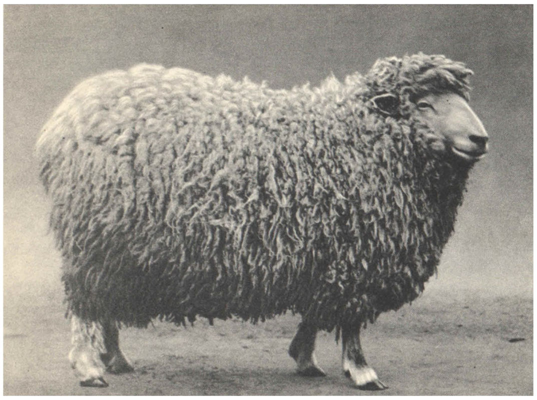

(**c**)

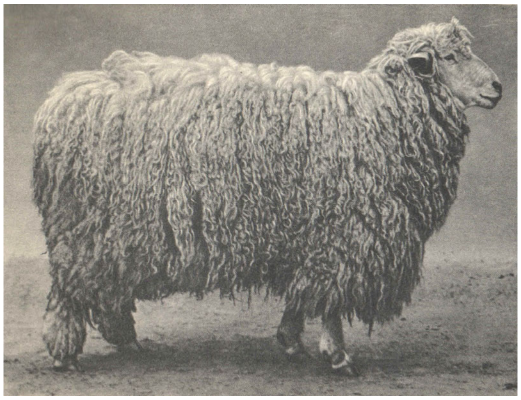

(**d**)

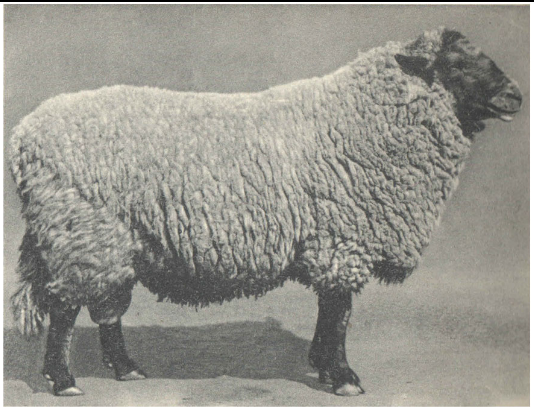

(**e**)

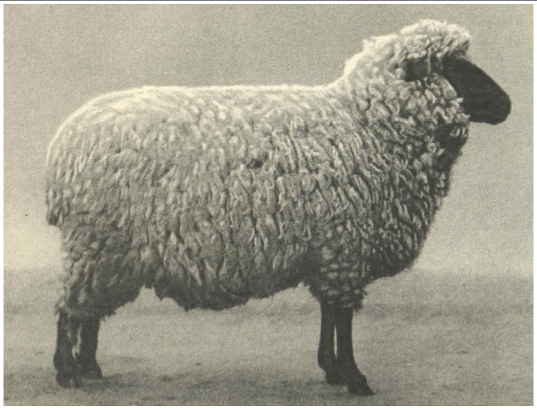

(**f**)

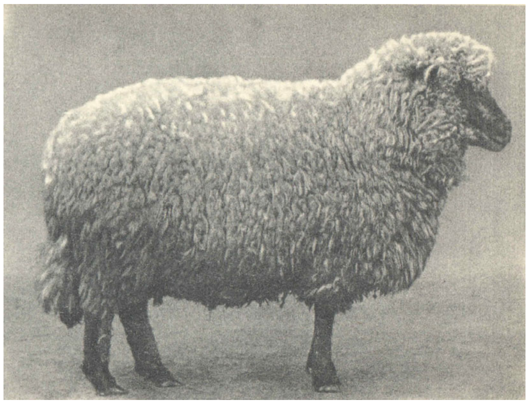

(**g**)

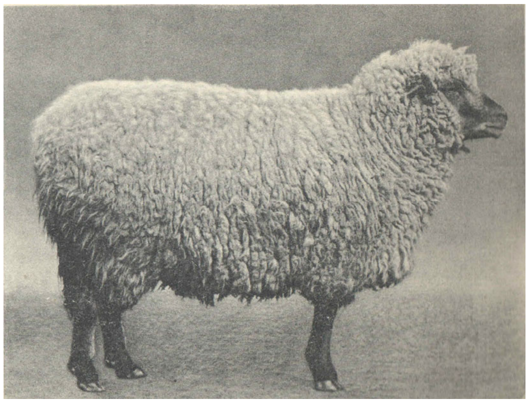

(**h**)

Figure S2. Examples of Soviet sheep breeds produced using British breeds and presented at the All-Union Agricultural Exhibition (AUAE): (**a**) Mikhnov × Lincoln crossbred ewe (year of birth, 1938; wool fineness, 44–46; body weight, 104 kg; AUAE breed champion, 1940) [19]; (**b**) Kuibyshev ram (year of birth, 1950; body weight, 152 kg; wool yield, 6.3 kg; wool length, 21 cm; wool fineness, 46; AUAE champion, 1954) [20]; (**c**) Pechora ram (year of birth, 1951; body weight, 94 kg; wool yield, 5.62 kg; wool length, 12 cm; wool fineness, 50; 1st degree AUAE certificate, 1954) [20]; (**d**) Russian Long-wool ram (year of birth, 1951; body weight, 126 kg; wool yield, 9.5 kg; wool length, 24 cm; wool fineness, 44; AUAE champion, 1954) [20]; (**e**) Lithuanian Black-headed ram (year of birth, 1949; body weight, 131 kg; wool yield, 6.2 kg; wool length, 11 cm; wool fineness, 56–50; 1st degree AUAE certificate, 1954) [20]; (**f**) Gorky ewe (year of birth, 1951; body weight, 79 kg; wool yield, 5.3 kg; wool length, 10 cm; wool fineness, 56; 2nd degree AUAE certificate, 1954) [20]; (**g**) Latvian Dark-headed ewe (year of birth, 1949; body weight, 102 kg; wool yield, 6 kg; wool length, 10.5 cm; wool fineness, 56–50; AUAE breed champion, 1954) [20]; (**h**) Estonian Black-headed ram (year of birth, 1952; body weight, 85 kg; wool yield, 4.8 kg; wool length, 11 cm; wool fineness, 56–50; 2nd degree AUAE certificate, 1954) [20].

References

1. Deikhman, E.K. *Organization of Work at a Sheep Farm*; OGIZ–Selkhozgiz: Moscow, Russia, 1947; 120p.
2. Ministry of Agriculture of the USSR. *All-Union Agricultural Exhibition of 1954*; Ministry of Agriculture of the USSR: Moscow, Russia, 1955; 118p.

Table S1. Origin data and phenotypic characteristics of selected British sheep breeds.

Name	Alias	Origin	Characteristics	References
		Breeds of England		
Border Leicester	—	cross of Dishley Leicester and Teeswaters or Cheviots produced Blucaps or Redlegs, 18th century; Redleg became Border Leicester in 1850	bred for meat; rare; large; hornless; erect ears; Roman nose; good mothering ability; fast growing, early maturing lambs; used for crosses; largest indigenous breed in the British Isles in the past	[4,5,6,7,9]
Clun Forest	—	Clun, England on the Welsh Border and surrounding forests of Shropshire; as early as 1803; crossed with Longmynd, Radnor and Shropshire sheep	bred for meat, milk and wool; maternal breed; medium-sized; good mothering ability; quality lambs; ability to finish off grass; adaptable to various climates; used for crosses	[1,4,7,10]
Dorset Horn	Dorset, Dorset Horned	dates back to 1693; crossed in Australia in the early 20th century with a variety of breeds to create Poll Dorset, exported back to UK and became more popular than horned type	bred for meat and wool; rare; horned, with large spirals in males; stocky; adaptable; prolific; slightly shorter gestation period, ability to breed out of season (3 crops of lambs in 2 years)	[1,4,7,9]; Figure S1d,e
Romney	Romney Marsh, Kent	Romney Marshes of Kent and Sussex, South East England, 13th century	bred for meat and wool; large; long-woolled; no horns; hardy; strong constitution; good health; adaptability; minimum management; excellent foragers; prime lambs; large fleece suitable for carpets	[1,2,4,6,7,]; Figure S1g
Suffolk	—	Bury St Edmunds area of Suffolk, Eastern England, 1810; developed by crossing Southdown rams and Norfolk Horn ewes	bred for meat; dominant, most used native terminal sire breed; lowland breed; large; high milk output, hard hooves and easy lambing traits in ewes; fast-growing quality lambs of good conformation	[1,2,4,5,6,7,]
Wiltshire Horn	—	Wiltshire, Southern England; descends from original sheep brought by Romans	bred for meat; originally local lowland breed; large; long legged; horned; sheds wool, not requiring shearing; low input, economical; easy management; relatively hardy; easy lambing; good mothering traits; good carcase shape and weight of lambs; used for crosses	[1,2,4,7,]
		Breeds of Scotland		
Blackface including Scottish Blackface type	Scottish Blackface: Blackfaced Highland, Kerry, Linton, Scotch Blackface, Scotch Horn, Scottish Highland, Scottish Mountain	UK, 12th century; Scottish Blackface: Scotland; shares origin with other horned sheep (Swaledale and Rough Fell)	bred for meat; horned; maternal hill breed; hardy; strong mothering ability; suited to extreme conditions	[1,2,7,11]; Figure S1a
Cheviot	—	Cheviot Hills between Scotland and England, as early as 1372; crossed with Merino imported into Berwick in 1480-1560; includes more than one subtype (e.g., South Country Cheviot)	bred for meat and wool; maternal hill breed; males sometimes horned; very hardy; good mothering ability; fast-maturing, quality lambs	[1,7,] Figure S1b,c
		Breeds of Wales		
Badger Face Welsh Mountain	Defaid Idloes, Badger Faced Welsh Mountain, Welsh Badger-faced	1st century; Welsh Mountain subtype	bred for meat; one of the oldest UK breeds; badger face; very hardy; strong mothering ability; fast growing lambs;	[7,10]

(of Torddu and Torwen types)			suited to upland and lowland conditions; good lambing percentage in upland conditions (160%)	
Balwen Welsh Mountain	—	19th century; almost extinct in 1946–1947, when 1 ram left	bred for meat; rare; horned males; small females; maternal hill breed; very hardy; strong mothering ability; suited to difficult conditions; few health problems; minimum supplementary feeding	[5,6,7,9,10]
Beulah Speckled Face	Epynt Hill & Beulah Speckled Face	hills of Epynt, Llanafan, Abergwesyn and Llanwrtyd Wells, Wales, >100 years ago; developed from mountain sheep with no crossing; Welsh Mountain subtype	bred for meat; no hornes; hardy upland sheep; thrive in harsh environments; used for crosses	[5,7,10]
Black Welsh Mountain	Defaid Mynydd Duon	Wales, 13rd century; Welsh Mountain subtype	bred for meat; small; polled ewes, horned males; maternal hill breed; hardy; strong mothering ability; suited to extreme conditions	[4,6,7,10]
Brecknock Hill Cheviot	Brecon Cheviot, Sennybridge Cheviot	Brecon Beacons, Wales, 400 years ago; descends from the early Border Cheviot, Scotland; distinct breed in the mid 19th century; Cheviot subtype	bred for meat; hill breed; broad-backed; polled females, males polled or horned; very hardy; long-lived; used as a crossing sire	[1,7,10]
Dollgellau Welsh Mountain	—	Welsh Mountain subtype	bred for meat and wool	[10]
Hill Radnor	—	originates from old Welsh tan-faced sheep of the Radnor Hills area of mid-Wales, 1911	bred for meat; rare; native hill breed; strong, heavy boned; curved horns in males that spiral outwards; hardy; thrive in harsh conditions; excellent mothering ability, 1.5 lambs a year; slow maturing lambs; good mutton producer	[1,2,7,9,10]
Improved Welsh Mountain	—	Welsh Mountain subtype	bred for meat and wool	[10]
Kerry Hill	—	originates from the village of Kerry in mid-Wales (area of England-Wales border), as early as 1809	bred for meat; native; proud stance; upright ears; no horns; sturdy; adaptable to lowland systems; good mothering ability	[7,10]
Llandovery Whiteface	Llandovery White Faced	Llandovery, Wales, bred for generations around Black Mountain of Carmarthenshire, with Llandovery Market being renowned for sales of breeding females	bred for meat; upland breed; larger in stature; males sometimes horned; hardy; good maternal quality; used for crosses	[7,10]
Llanwenog	—	Teifi Valley, West Wales, late 19th century; mixture of several breeds incl. Welsh Mountain, Clun Forest, Shropshire and extinct Llanllwni	bred for meat; rare; upland breed; medium sized; hardy; produce more lambs (usually twins); easy to manage; versatile; prolific; high quality and flavoured meat	[4,5,6,7,9,10]
Lleyn	Dafad Llŷn	Lleyn peninsula, North Wales, early 19th century; derived from crossing local Welsh sheep with Roscommon (a cross of indigenous Irish sheep and Dishley Leicester) exported from Ireland	bred for meat; maternal breed; medium sized; no horns; low maintenance; thrive on upland and lowland grazing; prolific; low disease risk; used for crosses	[5,6,7,10]
South Wales Welsh Mountain	—	—	bred for meat and wool	[10]
Talybont Welsh	Talybont Welsh Mountain	developed in Talybont, Usk valley, on Brecon Beacons, Llangynidr	bred for meat; one of larger Welsh Mountain breeds; maternal hill breed;	[7,10]

		Mountain and Black Mountains for several centuries	horned or polled; hardy; excellent mothering skills in harsh environments; heavier prime lambs	
Tregaron Welsh Mountain	—	—	bred for meat and wool	[10]
Welsh Hardy Speckled Face	—	—	bred for meat and wool	[10]
Welsh Mountain-Hill Flock	Hill Flock Welsh Mountain	Welsh hills of Glamorgan, Monmouthshire, Carmarthenshire and South Powys, 13th century	bred for meat; maternal hill breed; moderate length and height; males can be horned; economical and hardy; thrive in extreme environments; good versatility and survivability; excellent mothering ability	[7,10]

References

3. Youatt, W. *Sheep: Their Breeds, Management, and Diseases: To which is Added the Mountain Shepherd's Manual*; Baldwin and Cradock: London, UK, 1837.
4. Ryder, M.L. The history of sheep breeds in Britain. *Agric. Hist. Rev.* **1964**, *12*, 1–12, 65–82.
5. Ryder, M.L. A survey of European primitive breeds of sheep. *Ann. Genet. Sel. Anim.* **1981**, *13*, 381–418, doi:10.1186/1297-9686-13-4-381.
6. *World Watch List for Domestic Animal Diversity*, 3rd ed.; Scherf, B.D., Ed.; FAO: Rome, Italy, 2000.
7. Green, K. Shaggy sheep stories. *Ctry. Life* **2017**, *121*, 68–72.
8. The Natural Fibre Company. Meet the Animals. Available online: https://www.thenaturalfibre.co.uk/meet-the-animals (accessed on 3 February 2021).
9. National Sheep Association. Sheep Breeds. Available online: https://www.nationalsheep.org.uk/uk-sheep-industry/sheep-in-the-uk/sheep-breeds/ (accessed on 3 February 2021).
10. Schoenian, S. Border Cheviot. Sheep Breeds C. Sheep101.info. Available online: http://www.sheep101.info/breedsB.html#Cheviot (accessed on 3 February 2021).
11. Rare Breeds Survival Trust. Sheep watchlist. Available online: https://www.rbst.org.uk/Pages/Category/sheep-watchlist (accessed on 3 February 2021).
12. Beynon, S.E.; Slavov, G.T.; Farré, M.; Sunduimijid, B.; Waddams, K.; Davies, B.; Haresign, W.; Kijas, J.; MacLeod, I.M.; Newbold, C.J.; et al. Population structure and history of the Welsh sheep breeds determined by whole genome genotyping. *BMC Genet.* **2015**, *16*, 65, doi:10.1186/s12863-015-0216-x.
13. Clark, E.L.; Bush, S.J.; McCulloch, M.E.B.; Farquhar, I.L.; Young, R.; Lefevre, L.; Pridans, C.; Tsang, H.G.; Wu, C.; Afrasiabi, C.; et al. A high resolution atlas of gene expression in the domestic sheep (*Ovis aries*). *PLoS Genet.* **2017**, *13*, e1006997, doi:10.1371/journal.pgen.1006997.

Table S2. Native sheep breeds from Russia and former USSR developed using British breeds.

Breed/ Population	Maternal Breed/ Population	Paternal Breed	Period of Breeding	Distribution/ Population Size	Reference
			Long-wool sheep		
Mikhnov (improved)	local Mikhnov coarse-wool sheep well known in the 19th century	Lincoln, Cotswold, Oxford	beginning of the 20th century	central regions of Russia; lost in the second half of the 20th century	[22]; Figure S2a
Kuchugury (improved)	local Kuchugury coarse-wool sheep well known in the 19th century	Lincoln	beginning of the 20th century	central regions of Russia; 7019 animals in 1990; current number unknown; remained pure-bred animals are kept at private farms	[22]
Kuibyshev	Cherkasy coarse-wool sheep	Romney	1936–1948	central and middle part of Russia; 204,745 animals in 1990, 4455 animals in 2019	[22,24]; Figure S2b
Pechora population	local Northern coarse-wool sheep	Romney, Kuibyshev	1930–1960, lost in the 1990s and restored in the 2010s	Komi Republic, Russia; a single population of ~250 animals kept at the Pechora Breeding Station	[23]; Figure S2c
North Caucasian Meat-and-wool	Stavropol fine-wool sheep	Lincoln, Romney	1944–1961 (recognized in 1962)	southern regions of Russia; 1,298,302 animals in 2009, 12,654 animals in 2019	[22,24]
Tien-Shan	Précoce × local Kyrgyz fat-rumped crossbred sheep	Lincoln	1950–1966	Kyrgyzstan; 334,509 animals in 1990, 200,000 animals in 2002, currently at risk of extinction	[18,29]
Russian Long-wool	Mikhnov, Kuchugury and Northern coarse-wool sheep	Lincoln	1936–1978	central regions of Russia; 417,731 animals in 1990, 1400 animals in 2019	[22,24]; Figure S2d
Soviet Meat-and-wool (Caucasian type)	crosses of coarse-wool and fine-wool sheep	Lincoln, Russian Long-wool, North Caucasian Meat-and-wool	1950–1985	southern regions of Russia; 1,088,692 animals in 1990, 5410 animals in 2019	[22,24]
Soviet Meat-and-wool (Siberian type)	local fine-wool sheep, mainly the Altai breed	Lincoln, Romney	1960–1988	Ural and Western Siberia; current number unknown	[22]
			Short-wool sheep		
Lithuanian Black-headed	German Black-headed	Hampshire	1923–1934	Baltic countries, Russia; 9089 animals in Lithuania in 2016, 1956 animals in Russia in 2019	[18,24]; Figure S2e
Latvian Dark-headed	local short-tail sheep and their crosses with Merinos	Shropshire, Oxford	1923–1937 (herdbook established in 1940)	Baltic countries; 246,768 animals in the USSR in 1990, 34,566 animals in Latvia in 2013	[18,22]; Figure S2g
Estonian Black-headed	Northern short-tail sheep and their crosses	Shropshire	Second half of the 19th century (herdbook established in 1958)	Baltic countries; 9548 animals in Estonia in 2007	[18]; Figure S2h
Estonian White-headed	Northern short-tail sheep	Cheviot	Second half of the 19th century (herdbook established in 1958)	Baltic countries; 2151 animals in the USSR in 1990, 100,000 animals in Estonia in 2007	[18,22]
Gorky	local coarse-wool Northern short-tail prolific sheep	Hampshire	1936–1960	central regions of Russia; 47,890 animals in 1990, currently extinct	[22]; Figure S2f
Degeres Meat-and-wool	Précoce × local Kazakh fat-rumped sheep	Shropshire	1931–1980	Kazakhstan; 44,766 animals in 1990	[22]

References

18. FAO. Domestic Animal Diversity Information System (DAD-IS). Available online: http://www.fao.org/dad-is/en/ (accessed on 14 February 2021).
22. Semyonov, S.I.; Selkin, I.I. Sheep. In Animal Genetic Resources of the USSR; Dmitriev, N.G., Ernst, L.K., Eds.; Food and Agriculture Organization of the United Nations: Rome, Italy, 1989; Volume 65, pp. 154–271.
23. Kaneva, L.A.; Zharikov, Y.A.; Matyukov, V.S. Zootechnical characteristics of Pechora meat-wool semi-fine-fleece wool sheep. Agrarnaya Nauka Evro Severo Vostoka 2014, 5, 58–63, doi:10.30766/2072-9081.2014.42.5.58-63.
24. Yearbook on Breeding Work in Sheep and Goat Farming in the Farms of the Russian Federation (2019); All-Russian Research Institute of Animal Breeding: Moscow, Russia, 2020; 344p.
29. Luschihina, E.M. Sheep breed resources of Kyrgyzstan. In Collection of Scientific Papers Based on the International Coordination Congress of Scientists Sheep Breeders 2013; Collection of Proceedings of SNIIZHK; Stavropol Research Institute of Animal Husbandry and Food Production: Stavropol, Russia, 2013; pp. 67–80.

Supplementary Information S1

British sheep breeds outside the UK: examples from Russia and former USSR

British sheep breeds had been long attracted the attention of Soviet breeders due to their ability to combine high precocity and well-developed meat qualities with good wool productivity and excellent wool quality. The first import of British sheep to the USSR is dated back to 1923; a total of 152 thousand animals of various meat breeds were imported in 1926 to 1931 [21, 26]. However, purebred British sheep were not able to adapt to local climatic and feeding conditions in the primary sheep breeding and farming zones, i.e., in the South of the USSR with hot climate, highlands and often poor forage resources. Therefore, the main breeding strategy was to produce F_1 crosses and first-generation backcrosses (BC_1) between local and British meat breeds followed by strong selection of crossbred animals for meat and wool productivity [25].

Large-scale studies of 76 different crosses between 14 sheep breeds performed in 1925–35 showed the most efficiency when crossing local ewes with the Lincoln, Romney and Hampshire rams [27]. These had become the most widely used British sheep breeds to restore the semi-fine sheep husbandry in the USSR. The latter was almost completely destroyed by the Soviet famine of 1932–33 when the population of sheep and goats dramatically dropped from 107 million animals in 1928 to 37.3 million animals in 1933 [28].

In the Soviet time, the major goal of utilizing British sheep breeds was the improvement of local low-productive coarse-wool sheep and their unsystematic crossbreds with local fine-wool sheep. As reviewed by Semyonov and Selkin [22], the long-wool Lincoln and Romney rams were used for developing the Kuibyshev (herdbook being established in 1948), North Caucasian Meat-and-wool (1961), Tien-Shan (1966), Russian Long-wool (1978), and Caucasian and Siberian types of Soviet Meat-and-wool (1985, 1988) breeds. Besides, Lincoln, Cotswold and Oxford and Romney rams were used to improve the local Mikhnov, Kuchugury and Pechora sheep. The Hampshire, Shropshire, Oxford and Cheviot breeds contributed to creating Soviet semi-fine short wool breeds. Using Hampshire rams, the Lithuanian Black-headed (1934) and Gorky (1960) breeds were produced. Shropshire rams were employed in developing the Estonian Black-headed (1958) and Degeres Meat-and-wool (1980) breeds. Shropshire and Oxford rams were used to produce the Latvian Dark-headed breed (1940) and Cheviot rams to create the Estonian White-headed breed (1958) (Table S1, Figure S1). Thus, the British sheep breeds had a great impact on the development of the USSR genetic resources of semi-fine-wooled sheep.

Recent studies of the genetic architecture of semi-fine-wooled breeds of Russia [58] and Kyrgyzstan [59] using genome-wide SNP genotyping showed their relatedness to

British and North European sheep breeds. This was concordant with the breed origin history and elucidated presence of the ancestral British-specific genetic components in the current populations of Russia and Kyrgyzstan breeds.

Although numbers of the above Soviet breeds have declined significantly [18,24] (Table S1), most of them are still exploited in livestock production in Russia and former USSR countries or kept in gene pool herds as valuable national genetic resources [18,23,29].

References

18 FAO. Domestic Animal Diversity Information System (DAD-IS). Available online: http://www.fao.org/dad-is/en/ (accessed on 14 February 2021).

21. Ivanov, M.F. Sheep Farming. In Complete Works, in 7 Volumes; Greben, L.K., Ed.; Selkhozgiz: Moscow, Russia, 1964; Volume 4, 779p.
22. Semyonov, S.I.; Selkin, I.I. Sheep. In Animal Genetic Resources of the USSR; Dmitriev, N.G., Ernst, L.K., Eds.; Food and Agriculture Organization of the United Nations: Rome, Italy, 1989; Volume 65, pp. 154–271.
23. Kaneva, L.A.; Zharikov, Y.A.; Matyukov, V.S. Zootechnical characteristics of Pechora meat-wool semi-fine-fleece wool sheep. Agrarnaya Nauka Evro Severo Vostoka 2014, 5, 58–63, doi:10.30766/2072-9081.2014.42.5.58-63.
24. Yearbook on Breeding Work in Sheep and Goat Farming in the Farms of the Russian Federation (2019); All-Russian Research Institute of Animal Breeding: Moscow, Russia, 2020; 344p.
25. Kuleshov, P.N. Value of Merino and English Meat Breeds in Improving Sheep Farming in the USSR; Moscow Higher Zootechnical Institute: Moscow, Russia, 1926; 16p.
26. Kuleshov, P.N. Meat-and-Wool Sheep Breeding; Selkhozgiz: Moscow, Russia, 1933; 112p.
27. Glembotsky, Y.L.; Deikhman, E.K.; Esaulov, P.A. Breeding in Sheep Farming: Achievements in Developing New Sheep Breeds and Improving Existing Ones; Selhozgiz: Moscow, Russia, 1946; 151p.
28. Ostrovsky, A.V. Universal Reference Book on the History of Russia: With Tables, Diagrams and Dictionaries; Paritet: St. Petersburg, Russia, 2000; 384p.
29. Luschihina, E.M. Sheep breed resources of Kyrgyzstan. In Collection of Scientific Papers Based on the International Coordination Congress of Scientists Sheep Breeders 2013; Collection of Proceedings of SNIIZHK; Stavropol Research Institute of Animal Husbandry and Food Production: Stavropol, Russia, 2013; pp. 67–80.58 Deniskova, T.E.; Dotsev, A.V.; Selionova, M.I.; Kunz, E.; Medugorac, I.; Reyer, H.; Wimmers, K.; Barbato, M.; Traspov, A.A.; Brem, G.; et al. Population structure and genetic diversity of twenty-five Russian sheep breeds based on whole-genome genotyping. *Genet. Sel. Evol.* **2018**, *50*, 29. doi: 10.1186/s12711-018-0399-5.

59. Cinar, M.U.; Mousel, M.R.; Herndon, M.K.; Taylor, J.B.; White, S.N. Association of TMEM8B and SPAG8 with mature weight in sheep. Animals 2020, 10, 2391, doi:10.3390/ani10122391.
